# Forever Young(er): potential age-defying effects of long-term meditation on gray matter atrophy

**DOI:** 10.3389/fpsyg.2014.01551

**Published:** 2015-01-21

**Authors:** Eileen Luders, Nicolas Cherbuin, Florian Kurth

**Affiliations:** ^1^Department of Neurology, School of Medicine, University of California, Los AngelesLos Angeles, CA, USA; ^2^Centre for Research on Ageing Health and Wellbeing, Australian National UniversityCanberra, ACT, Australia

**Keywords:** aging, brain, gray matter, meditation, mindfulness, MRI, VBM

## Abstract

While overall life expectancy has been increasing, the human brain still begins deteriorating after the first two decades of life and continues degrading further with increasing age. Thus, techniques that diminish the negative impact of aging on the brain are desirable. Existing research, although scarce, suggests meditation to be an attractive candidate in the quest for an accessible and inexpensive, efficacious remedy. Here, we examined the link between age and cerebral gray matter re-analyzing a large sample (*n* = 100) of long-term meditators and control subjects aged between 24 and 77 years. When correlating global and local gray matter with age, we detected negative correlations within both controls and meditators, suggesting a decline over time. However, the slopes of the regression lines were steeper and the correlation coefficients were stronger in controls than in meditators. Moreover, the age-affected brain regions were much more extended in controls than in meditators, with significant group-by-age interactions in numerous clusters throughout the brain. Altogether, these findings seem to suggest less age-related gray matter atrophy in long-term meditation practitioners.

## Introduction

Life expectancy around the world has risen dramatically, with more than 10 years of life gained since 1970. While this demonstrates major advances in healthcare and public health, it also presents major challenges: The human brain starts to decrease in volume and weight from our mid-twenties onwards (Fotenos et al., 2005; Walhovd et al., 2011; Oh et al., 2014). This structural deterioration leads progressively to functional impairments and is accompanied by an increased risk of mental illness and neurodegenerative disease (Fotenos et al., 2005; Kooistra et al., 2014). With an aging population, the incidence of cognitive decline and dementia has substantially increased in the last decades. In this light, it seems essential that longer life expectancies do not come at the cost of reduced life qualities, so that individuals can spend their increased lifetime living as healthy and satisfying as possible. Naturally, this requires a better understanding of the pathological mechanisms leading to brain aging but also the identification of factors which are protective of cerebral health and particularly those that can have incremental effects across the lifespan. Much research has focused on the identification of risk factors, but relatively less attention has been turned to positive approaches aimed at enhancing cerebral health.

Meditation might be a possible candidate in the quest for such a positive approach as there is ample evidence for its beneficial effects for a number of cognitive domains, including attention, memory, verbal fluency, executive function, processing speed, overall cognitive flexibility as well as conflict monitoring and even creativity (Lutz et al., 2008, 2009; Colzato et al., 2012; Gard et al., 2014; Lippelt et al., 2014; Marciniak et al., 2014; Newberg et al., 2014). This wealth of cognitive studies did not only further support the idea that the human brain (and mind) is plastic throughout life but also lead to a number of relevant concepts and theories, such as that meditation is associated with an increasing control over the distribution of limited brain resources (Slagter et al., 2007) as well as with process-specific learning, rather than purely stimulus- or task-specific learning (Slagter et al., 2011). Nevertheless, studies exploring the actual brain-protective effects of meditation are still sparse. As recently reviewed (Luders, 2014), there are only three published studies examining if correlations between chronological age and cerebral measures are different in meditators and controls. The first study (Lazar et al., 2005) focused on cortical thickness in two predetermined^1^ brain areas. The second study (Pagnoni and Cekic, 2007) focused on whole-brain as well as voxel-wise gray matter. The third study (Luders et al., 2011) focused on fractional anisotropy, an indicator of white matter fiber integrity, in 20 predefined^2^ fiber tracts. The outcomes from all three studies seem to suggest that meditation may slow, stall, or even reverse age-related brain degeneration, as there were less pronounced negative correlations and even positive correlations in meditators compared to controls (for a more detailed summary see Luders, 2014).

To further expand this field of research, we set out to examine the link between age and brain atrophy using an approach similar to the one used in one of the aforementioned studies (Pagnoni and Cekic, 2007). However, while Pagnoni and Cekic collected valuable data in a relatively small sample of 25 subjects (12 meditators/13 controls) with a mean age in the thirties, the present study included a large sample of 100 subjects (50 meditators/50 controls) with a mean age in the fifties. Using the present sample spanning a wide age range (24–77 years), we calculated the group-specific age-related correlations and tested for significant group-by-age interactions with respect to whole-brain gray matter volumes (hereafter referred to as *global* gray matter) as well as voxel-wise gray matter volumes (hereafter referred to as *local* gray matter). We expected reduced negative correlations in meditation practitioners compared to age-matched control subjects.

## Methods

### Subjects and imaging

Our study included 50 meditation practitioners (28 men, 22 women) and 50 control subjects (28 men, 22 women). Meditators and controls were closely matched for chronological age, ranging between 24 and 77 years (meditators [mean ± SD]: 51.4 ± 12.8 years; controls [mean ± SD]: 50.4 ± 11.8 years). Meditators were recruited from various venues in the greater Los Angeles area. Years of meditation experience ranged between 4 and 46 years (mean ± SD: 19.8 ± 11.4 years). A detailed overview with respect to each subject's individual practice is provided in Supplementary Table 1. Brain scans for the control subjects were obtained from the International Consortium for Brain Mapping (ICBM) database of normal adults (http://www.loni.usc.edu/ICBM/Databases/). The majority (89%) of study participants was right-handed; six meditators and five controls were left-handed. Importantly, all subjects were scanned at the same site, using the same scanner, and following the same scanning protocol. Specifically, magnetic resonance images were acquired on a 1.5 Tesla Siemens Sonata scanner (Erlangen, Germany) using an 8-channel head coil and a T1-weighted magnetization-prepared rapid acquired gradient echo (MPRAGE) sequence with the following parameters: 1900 ms repetition time, 4.38 ms echo time, 15° flip angle, 160 contiguous sagittal slices, 256 × 256 mm field-of-view, 1 × 1 × 1 mm voxel size. All procedures pertaining to this study were reviewed and approved by UCLA's Institutional Review Board; all subjects gave their informed consent.

### Data processing and analyses

**Figure 1:**
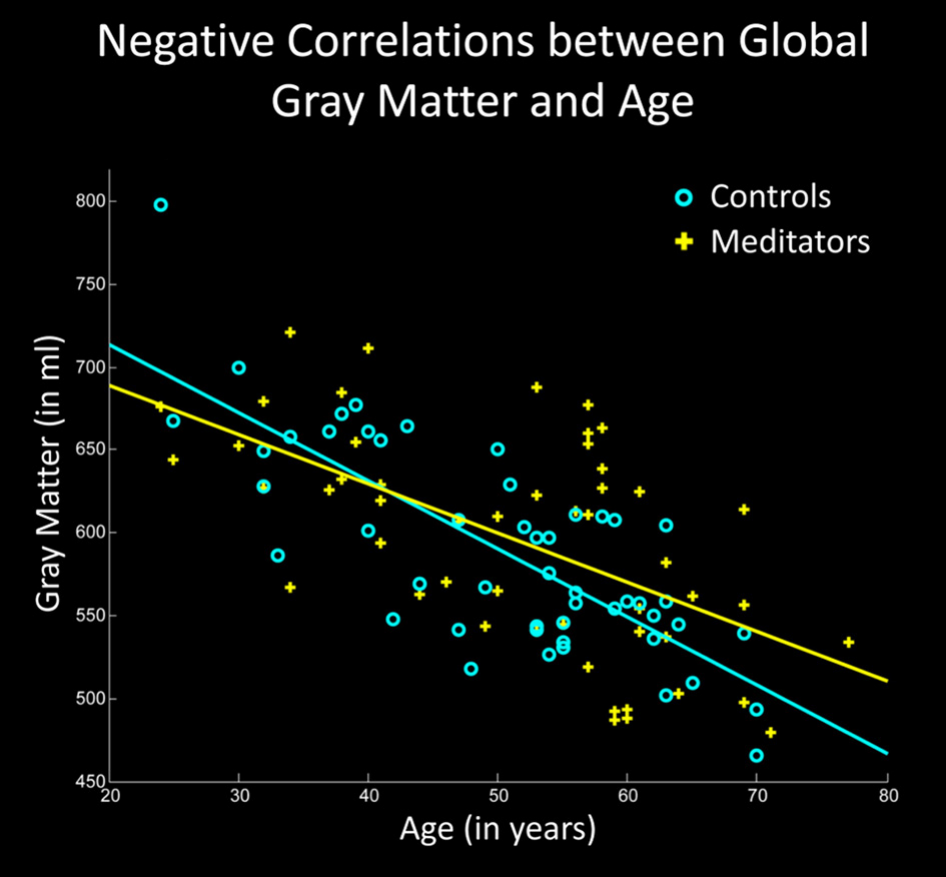
**Negative correlations between global gray matter and age**. The X-axis displays the chronological age (in years); the Y-axis displays the global gray matter volume (in ml). Note the less steep slope of the regression line in meditators (yellow) compared to controls (cyan).

All T1-weighted images were processed in Matlab (http://www.mathworks.com/products/matlab/) using SPM8 (http://www.fil.ion.ucl.ac.uk/spm) and voxel-based morphometry (VBM) standard routines as implemented in the VBM8 Toolbox (http://dbm.neuro.uni-jena.de/vbm.html), as previously described (Luders et al., 2013a). Briefly, images were corrected for magnetic field inhomogeneities (bias correction) and tissue-classified into gray matter, white matter, and cerebrospinal fluid (segmentation). Importantly, the tissue segmentation algorithm accounted for partial volume effects, which is crucial for the accurate estimation of tissue volumes (Tohka et al., 2004). To generate the input for the *global* gray matter analysis, we used the resulting gray matter partitions in their native dimensions and calculated the individual gray matter volumes (in ml) by summing up the voxel-wise gray matter content (across the entire brain) and multiplying it by voxel size. In order to characterize the group-specific direction and magnitude of age-related associations with respect to global gray matter, we first calculated the Pearson's correlations separately within meditators and controls, while removing the variance associated with sex (see Figure 1). Then, we tested if the correlations between age and global gray matter were significantly different between meditators and controls (group-by-age interaction), again, while co-varying for sex. All statistical analyses pertaining to global gray matter were conducted in Matlab using the Statistics Toolbox (http://www.mathworks.com/products/statistics/).

**Figure 2:**
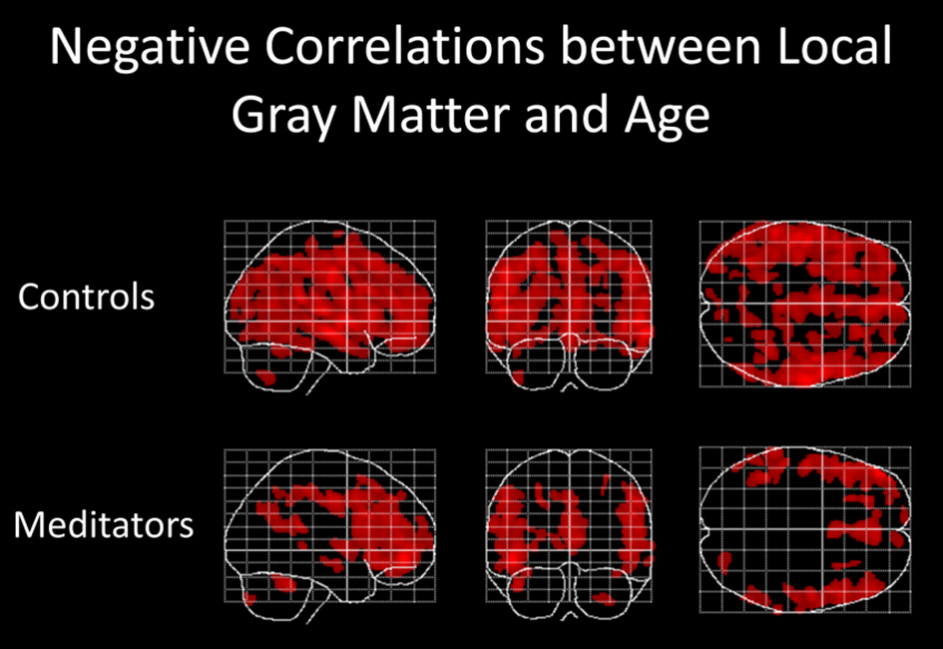
**Negative correlations between local gray matter and age**. Displayed are maximum intensity projections superimposed onto the SPM standard glass brain (sagittal, coronal, axial). Shown, in red, are significant negative age-related correlations within controls **(top)** and meditators **(bottom)**. Significance profiles are corrected for multiple comparisons via controlling the family-wise error (FWE) rate at *p* ≤ 0.05. Note the less extended clusters in meditators compared to controls.

**Figure 3:**
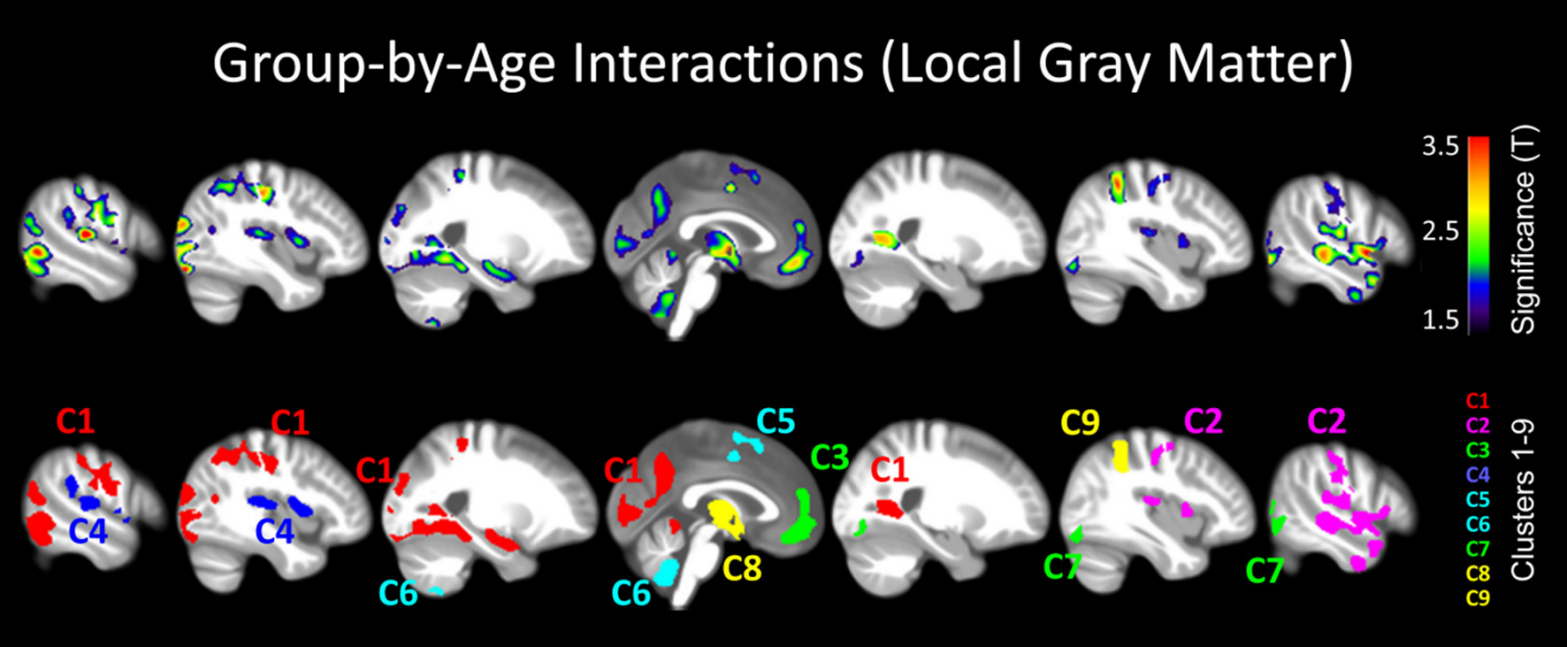
**Group-by-age interactions (local gray matter)**. The results are projected onto sagittal sections of the mean image derived from all subjects (*n* = 100). The clusters indicate areas where correlations between local gray matter and age are significantly different between meditators and controls (group-by-age interactions). Shown are clusters significant at *p* ≤ 0.05 with a spatial extent threshold of *k* ≥ 1039 voxels. **Top Row**: the different colors encode the T-statistic at the voxel level. **Bottom Row**: the different colors depict the nine clusters (C1–C9), as detailed in Table 1.

In parallel, to generate the input for the *local* gray matter analysis, we used the segmented gray matter partitions and normalized them spatially to the DARTEL template (provided with the VBM8 Toolbox) applying linear 12-parameter transformations and non-linear high-dimensional warping (Ashburner, 2007). The normalized gray matter segments were then multiplied by the linear and non-linear components derived from the normalization matrix (modulation) and convoluted with an 8 mm full-width-at-half-maximum (FWHM) Gaussian kernel (smoothing). These modulated, smoothed gray matter segments constitute the input for the subsequent statistical analyses: Mirroring the global gray matter analysis, we first calculated the correlations between age and local gray matter separately within meditators and controls in order to get a sense of the direction and extent of the age-related associations. For this purpose, we generated a series of maximum intensity projections within controls and meditators separately (see Figure 2). Then, we tested for significant group-by-age interactions (see Figure 3). For both analyses, group-specific correlations and group-by-age interactions, we removed the variance associated with sex and applied a significance threshold of *p* ≤ 0.05, corrected for multiple comparisons via controlling the family-wise error (FWE) rate. FWE-corrections resulted in a lack of significance clusters for the group-by-age interaction, but given that such interactions have been reported previously, we repeated the analysis without the rather conservative FWE-corrections. To discriminate real effects from spurious noise, we applied an appropriate spatial extent threshold (corresponding to the expected number of voxels per cluster) calculated according to the Gaussian random fields theory. All statistical analyses pertaining to local gray matter were conducted in Matlab using SPM (http://www.fil.ion.ucl.ac.uk/spm).

## Results

### Global gray matter

Examining the link between age and *whole-brain* gray matter, we observed a significant negative correlation in controls (*p* < 0.001) as well as in meditators (*p* < 0.001), suggesting age-related gray matter decline in both groups. However, as shown in Figure 1, the slopes of the regression lines were considerably steeper in controls than in meditators. Moreover, the group-specific correlation coefficients were higher in controls (*r* = −0.77) than in meditators (*r* = −0.58). The group-by-age interaction was highly significant (*p* = 0.003), altogether suggesting less age-related gray matter decline in meditators than in controls.

### Local gray matter

Examining the link between age and *voxel-wise* gray matter, significant negative correlations were evident in controls (*p* < 0.05, FWE-corrected) as well as in meditators (*p* < 0.05, FWE-corrected), suggesting age-related gray matter decline in both groups. However, as shown in Figure 2, age-affected brain regions were much more extended in controls than in meditators. In other words, echoing the global gray matter effect, the age-related decline of local gray matter was less prominent in meditators. Significant positive correlations were absent in both groups.

When mapping local group-by-age interactions applying a cluster size minimum of 1039 voxels (i.e., the expected number of voxels per cluster calculated according to the Gaussian random fields theory), we revealed nine significance clusters (C1–C9), as illustrated in Figure 3 and also Table 1. The largest cluster (C1) contains 20,019 voxels (corresponding to 68 ml of gray matter) traveling from the left hippocampus / amygdala posteriorly toward the left and right medial and left lateral occipital cortex, and then anteriorly toward the left medial and left lateral parietal cortex, from which it further expands toward the left central sulcus. The significance maximum of C1 is located at *x* = −42; *y* = −84; *z* = −17 (MNI space). Table 1 provides the details for all significance clusters (C1–C9) including the number of voxels, the cluster volume, the *T*-value and the MNI coordinates of the significance maximum, as well as the brain regions affected.

**Table 1:** **Cluster-specific details for significant group-by-age interactions (local gray matter)**.

**Cluster**	**Number of voxels**	**Volume (ml)**	**Significance maximum (*T*)**	**Significance maximum (*x, y, z*)**	**Brain regions**
**C1**	20,019	67.56	3.89	−42, −84, −17	Hippocampus/amygdala (L)
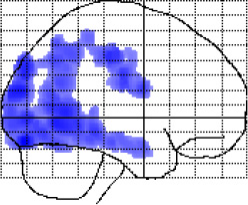					Medial (B) lateral (L) occipital cortex
					Medial (L) lateral (L) parietal cortex
					Posterior cingulate (L)
					Central sulcus (L)
**C2**	8,539	28.82	3.59	60, −4, −12	Sylvian F. w/operculum + insula (R)
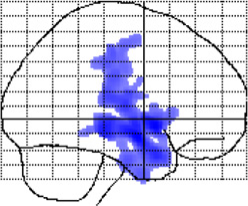					Lateral temporal cortex (R)
					Inferior parietal cortex (R)
					Central sulcus (R)
**C3**	2,986	10.08	2.99	2, 48, −12	Orbital gyrus (B)
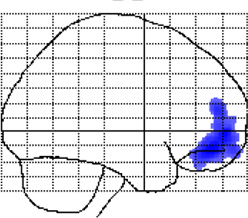					Anterior cingulate gyrus (B)
					Medial superior frontal gyrus (B)
**C4**	2,825	9.53	3.91	−52, −24, 10	Sylvian F. w/operculum + insula (L)
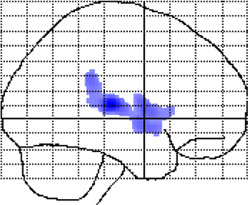					Temporo-parietal junction (L)
**C5**	1,856	6.26	3.19	−6, −4, 51	Mid cingulate gyrus (B)
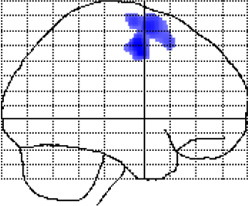					Medial superior frontal (B)
**C6**	1,542	5.20	2.70	−18, −61, −60	Cerebellum (B)
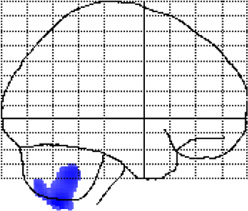					
**C7**	1,368	4.62	3.25	51, −75, −12	Lateral occipital cortex (R)
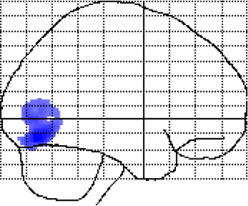					
**C8**	1,360	4.59	3.47	2, −4, −2	Hypothalamus (B)
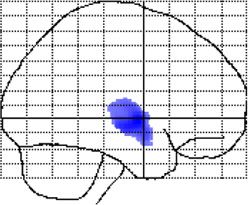					Medial thalamus (B)
**C9**	1,131	3.82	3.42	38, −45, 49	Lateral parietal cortex (R)
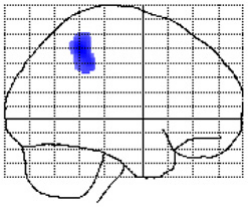					

*L, left hemisphere; R, right hemisphere; B, both hemispheres*.

## Discussion

We investigated the link between chronological age and gray matter in a large sample of long-term meditators and control subjects closely matched on age and sex. We observed that the age-related gray matter loss was less pronounced in meditators than in controls, both globally and locally. As summarized recently (Luders, 2014), there are only a few previous studies that were directed at exploring age-related brain atrophy in the framework of meditation (Lazar et al., 2005; Pagnoni and Cekic, 2007; Luders et al., 2011).

### Correspondence with prior research

In terms of the specific methods applied and cerebral features analyzed, our current analyses are most comparable to those done by examining gray matter. With respect to *global* gray matter, Pagnoni and Cekic (2007) reported a trend for a significant group-by-age interaction “with an estimated rate of change of −4.7 ml/year for the control group vs. +1.8 ml/year for the meditators group” (Pagnoni and Cekic, 2007). Our significant group-by-age interaction with respect to *global* gray matter seems to confirm these prior findings. Interestingly, however, while the aforementioned study (Pagnoni and Cekic, 2007) exposed a marginally significant negative correlation in the control group (*r* = −0.54) and a non-significant positive correlation in the meditation group (*r* = 0.006), our study revealed significant negative correlations between age and gray matter both in controls (*r* = −0.77) and in meditators (*r* = −0.58). The lack of positive correlations in meditators and the comparably stronger age-related decline in controls might be attributable to our considerably older cohort (with a mean age in the early-fifties), contrasting Pagnoni and Cekic's relatively young sample (with a mean age in the mid-thirties). Similarly, different mean ages—but also slightly different significance and spatial extent thresholds—might account for diverging findings between the two studies with respect to *local* gray matter: while Pagnoni and Cekic (2007) detected one significant cluster in the region of the putamen, the current study detected nine significant clusters spread throughout the entire brain (albeit none of them in the putamen).

The nine significance clusters, indicating different age-related correlations in meditators, are spanning large areas of the brain and include several structures where prior studies—although not focusing on aging effects *per se*—had revealed meditation effects, either as cross-sectional group differences and/or as longitudinal changes. For example, resembling the spatial location of previous observations, we detected significant group-by-age interactions within the left hippocampus [C1] (Holzel et al., 2011; Luders et al., 2013a,b), left and right insula [C2, C4] (Lazar et al., 2005; Holzel et al., 2008; Luders et al., 2012), left posterior cingulate gyrus [C1] (Holzel et al., 2011), right anterior cingulate gyrus [C3] (Grant et al., 2010, 2013), left and right superior frontal lobe, including precentral gyrus and central sulcus [C1, C2, C3, C5] (Lazar et al., 2005; Luders et al., 2009, 2012; Grant et al., 2013; Kang et al., 2013; Kumar et al., 2014), left and right inferior frontal lobe, including orbital gyrus [C3] (Luders et al., 2009; Vestergaard-Poulsen et al., 2009; Kang et al., 2013), left and right parietal lobe, including supramarginal gyrus, angular gyrus, and secondary somatosensory cortex [C1, C2, C4, C9] (Grant et al., 2010, 2013; Leung et al., 2013), right middle/inferior temporal cortex [C2] (Kang et al., 2013), left temporo-parietal junction [C4] (Holzel et al., 2011), right thalamus [C8] (Luders et al., 2009), as well as left and right cerebellum [C6] (Vestergaard-Poulsen et al., 2009; Holzel et al., 2011).

### Possible underlying mechanisms

In general, engaging the brain in intense mental activities has been suggested to stimulate dendritic branching and/or synaptogenesis (Greenwood and Parasuraman, 2010; Birch et al., 2013). These micro-anatomical changes might manifest on the macro-anatomical level as increased gray matter. Over time, such activity-induced gray matter gain may “mask” the gray matter loss that is normally observed in aging. In other words, the potential meditation-induced tissue increase might counteract the normal age-related decrease. In support of this stream of thought, evidence for increases in cerebral gray matter due to meditation has been provided (Holzel et al., 2011), where significant effects were detected within the hippocampus, posterior cingulate cortex, temporo-parietal junction, and cerebellum (i.e., all regions where our study also revealed significant effects; see clusters C1, C4, and C6). The fact that we detected significant group-by-age interactions in several additional regions might be attributable to our unique study population, which included expert meditators with a mean practice of close to 20 years, rather than meditation-naïve participants as examined in the aforementioned study (Holzel et al., 2011). Unfortunately, due to feasibility constraints, there is still a lack of longitudinal studies exploring the long-term effects of meditation.

An alternative (or complementary) mechanism to practice-induced gray matter *gain* might be practice-accompanying gray matter *conservation* over time (i.e., an actual deceleration of the gray matter loss itself). For example, meditation might conserve cerebral gray matter by reducing stress levels and thus modulating the potentially harmful effects of immune response genes expression (Irwin and Cole, 2011), HPA axis hyperactivity (McEwen, 2008), down-regulation of neurogenesis (Varela-Nallar et al., 2010), activation of pro-inflammatory processes and the production of reactive oxygen species (Swaab et al., 2005). Direct or indirect effects of stress reduction might manifest, especially in regions that are known to be particularly vulnerable against stress (e.g., the hippocampus; see cluster C1) and/or directly involved in the regulation of stress (e.g., the hypothalamus; see cluster C8). As an alternative to this stress-related mechanism, tissue preservation might also be the result of better health in general, perhaps a consequence of healthier habits related to eating, sleeping, working, physical exercise, and/or resulting from higher levels of (self-)awareness, intelligence, socioeconomic status, etc. However, given that none of these aforementioned factors has been systematically assessed for the entire sample, all this is merely conjecture. On this note, we also wish to emphasize that, given the cross-sectional design of our study, it is impossible to draw any clear causal inferences. In addition to the factors discussed above, the diminished age-related tissue loss as well as the meditation practice itself may be a consequence of certain personal traits and/or practice-promoting circumstances. For example, in order to keep meditating for close to 20 years, individuals need to possess a minimum level of discipline and commitment, a well-organized life that allows them the spare time, an awareness of the possibility to control their own life, perhaps even a calm nature to begin with. Clearly, not everyone has these traits, desires, and possibilities, and thus there might be a selection bias in our sample of long-term meditators. Future studies may thus further advance this field of research by capturing (and accounting for) characteristics unique to meditation samples.

### Conclusion and additional implications for future research

Altogether, our findings seem to add further support to the hypothesis that meditation is brain-protective and associated with a reduced age-related tissue decline. Nevertheless, it is important to acknowledge that the observed effects may not only be a consequence of meditating but also of other factors allowing for (or accompanying) a successful long-term practice. Moreover, given the cross-sectional nature of the present data with explicit focus on gray matter, further research—ideally using longitudinal data and perhaps exploring additional cerebral attributes—is necessary to establish the true potential of meditation to maintain our aging brains. Along these lines, future studies may also want to consider exploring possible differential effects of various meditation styles in the framework of brain aging. Similarly, as previously mentioned (Luders, 2014), it may be worthwhile to determine what constitutes the critical amount of meditation—preferably not only in terms of the number of practice hours or years in total, but also with respect to the length, frequency, and regularity of individual practice sessions—in order to accomplish desirable effects. In parallel, it remains to be defined what “desirable” actually means (for whom and in which context). Furthermore, given that the vast majority of older adults experience at least some deterioration in cognitive function, it seems valuable to extend purely anatomical analyses to investigations of cognitive abilities and decline. Knowing if (and how) the actual preservation of brain tissue is related to the preservation of mental skills, will add crucial insights to this emerging, but still understudied, research field “at the intersection of gerontology and contemplative sciences” (Gard et al., 2014). Accumulating scientifically solid evidence that meditation has brain (and mind) altering capacities might, ultimately, allow for an effective translation from research to practice, not only in the framework of healthy aging, but also pathological aging, such as is evident in mild cognitive impairment or Alzheimer's disease.

### Conflict of interest statement

The authors declare that the research was conducted in the absence of any commercial or financial relationships that could be construed as a potential conflict of interest.

## Footnotes

^1^The two areas were defined based on outcomes of a preceding analysis comparing meditators and controls with respect to cortical thickness: significant group differences were observed within the right insula and right frontal cortex.

^2^The twenty tracts were defined based on applying a white matter tractography atlas, which includes the following tracts: anterior thalamic radiation, corticospinal tract, uncinate fasciculus, inferior fronto-occipital fasciculus, inferior longitudinal fasciculus, superior longitudinal fasciculus (two sections: main/temporal component), cingulum (two sections: cingulate gyrus/hippocampus), as well as forceps major and forceps minor.
