# Supplementary material for: Forever Young(er): potential age-defying effects of long-term meditation on gray matter atrophy
Source: Front Psychol. 2015 Jan 21;5:1551. doi: 10.3389/fpsyg.2014.01551 (PMC4300906; doi:10.3389/fpsyg.2014.01551)
Supplement: Supplementary file 1 [file Table1.DOC]

**Supplementary Table 1.** Subject-specific meditation practices

| **M** | **Experience (in years)** | **Frequency**  **(times per week)** | **Duration**  **(minutes per session)** | **Meditation Style**  **(self-reported)** |
| --- | --- | --- | --- | --- |
| 1 | 4 | 4 | 30 | Shamatha, Vipassana |
| 2 | 5 | 7 | 10 | Shamatha |
| 3 | 6 | 5-6 | 15 | [not specified] |
| 4 | 7 | 3 | 40 | Zen |
| 5 | 7 | 7 | 60 | Kriya |
| 6 | 7 | 3-4 | 60 | Vipassana |
| 7 | 7 | 7 | 45 | Shamatha, Vipassana |
| 8 | 7 | 7 | 120 | Vipassana |
| 9 | 9 | 7 | 60 | Tibetan Buddhist Meditation |
| 10 | 9 | 3 | 40 | Vipassana |
| 11 | 10 | 7 | 60 | Shamatha, Vipassana, Zen |
| 12 | 10 | 3 | 20 | Kriya |
| 13 | 10 | 7 | 60 | Vipassana |
| 14 | 12 | 1 | 40 | [not specified] |
| 15 | 12 | 7 | 240 | Shamatha |
| 16 | 12 | 7 | 15 | [not specified] |
| 17 | 13 | 5-6 | 60 | Zen |
| 18 | 14 | 1-2 | 30 | Raja Yoga Meditation |
| 19 | 15 | 7 | 30 | Zen |
| 20 | 15 | 7 | 30 | Shamatha, Vipassana |
| 21 | 15 | 7 | 15 | [not specified] |
| 22 | 15 | 7 | 45 | Vipassana |
| 23 | 16 | 3-5 | 90 | Zen |
| 24 | 16 | 7 | 30 | [not specified] |
| 25 | 16 | 5-6 | 30 | Vipassana |
| 26 | 16 | 7 | 60 | Vipassana, Zen |
| 27 | 17 | 7 | 30 | Vajrayana |
| 28 | 18 | 6 | 30 | Mindfulness Meditation |
| 29 | 19 | 7 | 120 | Vipassana |
| 30 | 20 | 7 | 45 | Vipassana |
| 31 | 21 | 7 | 40 | Vipassana |
| 32 | 21 | 7 | 60 | Vipassana |
| 33 | 22 | 7 | 45 | Buddhist Meditation |
| 34 | 22 | 7 | 60 | [not specified] |
| 35 | 23 | 7 | 35 | Vipassana |
| 36 | 25 | 3 | 30 | Vipassana |
| 37 | 28 | 7 | 120 | Dzogchen, Vipassana |
| 38 | 30 | 3 | 30 | Zen |
| 39 | 31 | 7 | 60 | Sadhana, Shamatha, Vipassana, |
| 40 | 31 | 7 | 60 | Vipassana, Zen |
| 41 | 32 | 7 | 60 | Dzogchen |
| 42 | 33 | 7 | 60 | Vipassana |
| 43 | 36 | 6 | 20 | Zen |
| 44 | 36 | 7 | 45 | Dzogchen, Mahamudra, Vipassana |
| 45 | 38 | 1 | 60 | [not specified] |
| 46 | 38 | 3 | 90 | Chenrezig |
| 47 | 38 | 7 | 150 | Dzogchen, Vajrayana |
| 48 | 41 | 3 | 60 | Shamatha, Vipassana |
| 49 | 41 | 1 | 60 | [not specified] |
| 50 | 46 | 7 | 45 | Kundalini |

M: Meditators 1-50
